# The Role of Somatic Mutations in Ischemic Stroke: CHIP’s Impact on Vascular Health

**DOI:** 10.3390/neurolint17020019

**Published:** 2025-01-27

**Authors:** Aiman Kinzhebay, Amankeldi A. Salybekov

**Affiliations:** Qazaq Institute of Innovative Medicine, Regenerative Medicine Division, Cell and Gene Therapy Department, Astana 010000, Kazakhstan; a.kinzhebay@qaziim.kz

**Keywords:** stroke, CHIP, somatic mutation, vascular dysfunction, ischemic stroke, atherosclerosis

## Abstract

Clonal hematopoiesis of indeterminate potential (CHIP) is increasingly recognized as a significant contributor to ischemic stroke and other cardiovascular diseases due to its association with somatic mutations in hematopoietic cells. These mutations, notably in genes like *DNMT3A*, *TET2*, and *JAK2*, induce pro-inflammatory and pro-atherosclerotic processes, promoting vascular damage and stroke risk. With the prevalence of CHIP rising with age, its presence correlates with higher mortality and morbidity rates in ischemic stroke patients. This article explores the mechanisms through which CHIP influences vascular aging and stroke, emphasizing its potential as a biomarker for early risk stratification and a target for therapeutic intervention. The findings highlight the necessity of integrating CHIP status in clinical evaluations to better predict outcomes and personalize treatment strategies in stroke management.

## 1. Introduction

**Figure 1:**
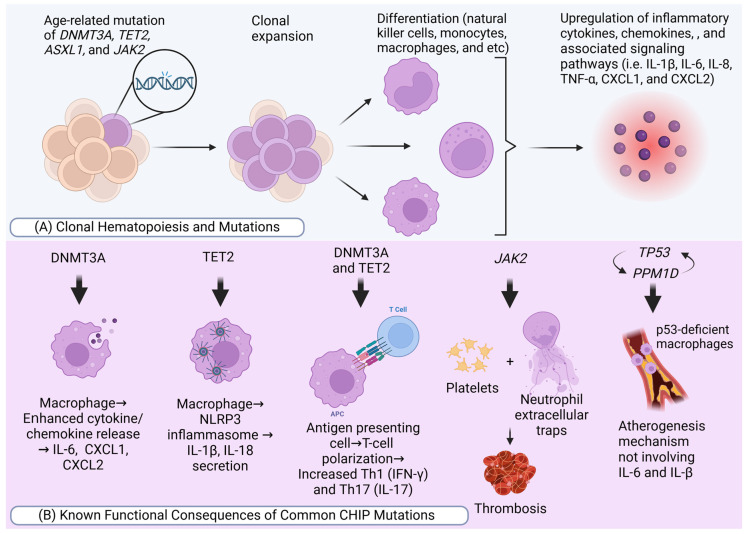
CHIP mutation and stroke. Created in BioRender©.

The World Health Organization defines clonal hematopoiesis (CH) as a population of cells derived from a mutated multipotent stem or progenitor cell with a growth advantage, occurring without unexplained cytopenias, hematologic cancers, or other clonal disorders [1] (Figure 1A). The incidence of CH increases with age and has been linked to higher overall mortality, cardiovascular diseases (CVDs), and myeloid malignancies [2]. Clonal hematopoiesis of indeterminate potential (CHIP) refers specifically to CH with somatic mutations in myeloid malignancy-associated genes, detectable at a variant allele fraction (VAF) of ≥2% in blood or bone marrow, in individuals without a diagnosed hematologic disorder or unexplained cytopenia [3].

CH was initially observed in the 1960s through studies on mosaic X chromosome activity [4], and further understanding was gained with advancements in large-scale sequencing, revealing recurrent genetic mutations associated with this phenomenon. These cytogenetic studies of chronic myeloid leukemia showed that hematologic malignancies have a clonal origin [4]. In the 1990s, studies of non-random X-chromosome inactivation in healthy women suggested that clonal hematopoiesis could be a common aspect of aging, but the specific genetic lesions were unidentified, leaving the idea controversial [4]. The term CHIP achieved prominence a decade ago after the publication of a hallmark study by Jaiswal et al. [2]. The proposal to redefine premalignant and cancerous conditions and introduce the term CHIP was based on studies showing that cancer-causing mutations in clonal hematopoiesis-associated genes (e.g., *DNMT3A*, *TET2*, *ASXL1*) can also occur in individuals without related disorders, such as acute myeloid leukemia or myelodysplastic syndrome [3]. These mutations are detectable in people with normal blood counts and no apparent disease, but they increase the risk of hematological malignancies and higher mortality [3].

Notably, CHIP has been increasingly recognized as a major risk factor for stroke, due to the pro-inflammatory effects of mutated hematopoietic cells [5]. These mutations can accelerate vascular aging and promote atherosclerosis, two critical pathways involved in stroke pathogenesis [5]. The identification of specific CHIP mutations also holds promise for personalized medicine, providing opportunities to tailor interventions based on an individual’s genetic risk profile. For instance, targeting inflammatory pathways or adjusting treatment plans according to CHIP mutation profiles is becoming a focus of precision medicine. Additionally, CHIP research has catalyzed the development of therapies targeting specific pathways, such as inflammation inhibitors or drugs designed to reduce clonal expansion. These advancements are paving the way for targeted treatments that can mitigate the risks associated with CHIP mutations, offering significant potential for improving patient outcomes. This growing evidence highlights the need for further study into how CHIP mutations contribute to stroke pathogenesis and how they can be used as predictive biomarkers.

## 2. Methods

To gather relevant information, guidelines, studies, and research papers associated with “somatic mutation”, “clonal hematopoiesis of indeterminate potential (CHIP)”, “clonal hematopoiesis”, “ischemic stroke”, “cardiovascular diseases”, and “stroke” were identified through searches on PubMed, Google Scholar, Scopus, and Cochrane Library databases. Reference lists of selected studies were also manually reviewed. Only guidelines, studies, and research articles available in English were included in the analysis. Studies were excluded if they (i) were not in English; (ii) consisted of conference abstracts, notes, letters, case reports, or animal studies; or (iii) were duplicate entries.

## 3. Emerging Mechanisms of Clonal Hematopoiesis in Stroke Pathogenesis

Newer, more sensitive assays have broadened the understanding of CH. Using methods capable of detecting mutations with VAFs below 2%, CH has been identified in over half of individuals older than 70 [6]. Furthermore, whole-exome sequencing has shown that CH can affect both myeloid and lymphoid driver genes, revealing a more comprehensive spectrum of CH that includes lymphoid CH (L-CH) [6]. These findings underscore the evolving definition of CH and its dependence on the scope and sensitivity of detection methods, which have also revealed key demographic trends. For instance, detectable somatic mutations are rare in individuals younger than 40 but increase significantly with age, reflecting a strong age-related pattern in CH prevalence [6] Studies find CH mutations in 6% of individuals aged 60–69, 12% for ages 70–89, and 20% of those aged 90 and older [6]. According to another report, there is a 9.5% prevalence in individuals aged 70–79, 11.7% in those aged 80–89, and for 90–108-year-olds, it is 18.4% [2]. Despite these variations, both studies highlight the commonality of CH in aging populations and its strong association with increased risks of CHIP-associated cardiovascular and hematologic conditions, emphasizing its clinical relevance [2,6].

Initially, CH was mostly known as a risk factor for myeloid malignancies. However, the seminal 2014 article revealed the increased mortality among individuals with CHIP mutations, which could not be fully explained by the risk of hematologic cancers [2]. This increased risk was proposed to be linked to other diseases such as coronary heart disease (CHD) and ischemic stroke (IS). The exact mechanism of how CHIP mutations cause those CVDs is not fully studied, but it is an actively developing direction in research. One potential explanation is that somatic mutations in hematopoietic cells may affect immune cell function, leading to the development of conditions such as atherosclerosis and even non-cardiovascular diseases like type 2 diabetes. Both CVDs and diabetes are chronic inflammatory conditions that frequently coexist, likely due to shared underlying inflammatory processes. The presence of systemic inflammation may exacerbate both conditions, highlighting the potential role of CHIP-mediated immune dysregulation in their comorbidity [2]. The relationship between CHIP and inflammatory diseases can be likened to a “chicken-and-egg” analogy, as it remains unclear whether CHIP drives the progression of inflammatory diseases or if chronic inflammation promotes the expansion of CHIP clones [7].

In a fixed-effects meta-analysis [5], it was found that individuals with any CHIP mutation had a 14% increased risk of total stroke (95% CI, 1.03–1.27; *p* = 0.01), particularly driven by a 24% increase in the odds of hemorrhagic stroke (HS) (95% CI, 1.01–1.51]; *p* = 0.04), such as subarachnoid hemorrhage (SAH) [5]. Restricting the analysis to individuals with a variant allele fraction greater than 10% did not significantly change these associations, with a continued increased risk for total stroke (18%, [95% CI, 1.01–1.51]; *p* = 0.04). The association between CHIP and IS was weaker (11%, [95% CI, 0.98–1.25]; *p* = 0.10), with a stronger connection to small vessel stroke (SVS) [5]. The study suggests that CHIP may contribute to cerebral small vessel disease, which is linked to both IS and intracerebral hemorrhage (ICH). The discussion also addresses the potential mechanisms involving inflammatory pathways that might link CHIP to aneurysm formation and vascular fragility, particularly in older individuals. Moreover, in individuals over the age of 80, the presence of CHIP was more strongly associated with ICH (HR 1.84, *p* = 0.01). In the second cohort, there was a higher prevalence of cerebral aneurysms and nontraumatic SAH among individuals with CHIP [5].

### 3.1. Vascular Aging and Endothelial Dysfunction

**Figure 2:**
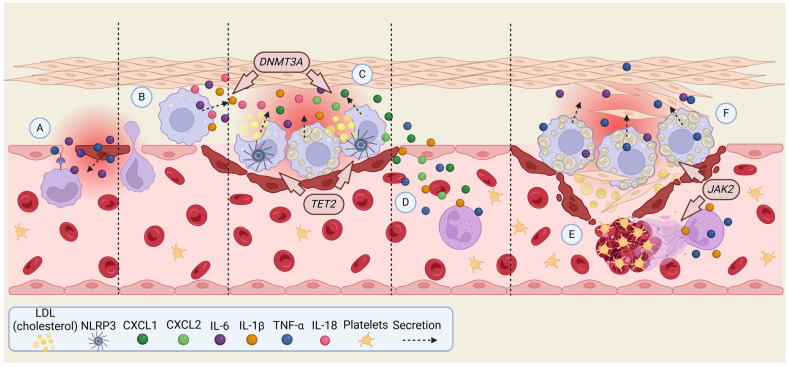
The general mechanism of CHIP-mediated atherogenesis and plaque rupture leading to stroke, with the inclusion of some contributing mutations. (**A**) IL-6 and TNF-α stimulate vascular permeability and expression of adhesion molecules to attract monocytes. (**B**) Monocytes exit the permeable vessel via diapedesis and turn into macrophages. (**C**) Macrophages phagocyte LDL and turn into foam cells and secrete cytokines. IL-18 activates NLRP3 inflammasome, which amplifies cytokine release. (**D**) CXCL1 and CXCL2 released by macrophages recruit additional monocytes and neutrophils to the site of inflammation. (**E**) IL-1β and TNF-α drive NET formation near the ruptured plaque leading to blood clotting. (**F**) Foam cells, smooth muscle, and other debris attracted by cytokines form a plaque that later raptures. Created in BioRender©.

Experimental studies have demonstrated the causal role of CHIP in cardiovascular disease, with specific mutations driving inflammation, as was mentioned above, and promoting atherogenesis (Figure 1B). For example, *TET2* deficiency in mice leads to larger atherosclerotic plaques and increased secretion of IL-1β, mediated by the NLRP3 inflammasome [8]. Similarly, *JAK2^V617F^* mutations enhance macrophage-driven plaque formation through the AIM2 inflammasome, leading to a more pronounced necrotic core in atherosclerotic plaques [9]. This means plaque-forming macrophages more readily engulf *JAK2^V617F^* erythrocytes (erythrophagocytosis), which subsequently impedes the macrophages’ ability to clear apoptotic cells (efferocytosis) [10]. These studies highlight key inflammatory pathways involving IL-1β and IL-6 as central to the development of atherosclerosis in CHIP carriers (Figure 2). Additionally, CHIP mutations have been implicated in atherosclerosis across the arterial system, including PAD. Mutations in *TP53*, for example, result in the accumulation of defective macrophages, promoting atherosclerosis via mechanisms distinct from the IL-1β and IL-6 pathways. This connection between CHIP and widespread arterial disease extends beyond the coronary system to the cerebral, renal, and mesenteric vasculature [11,12]. In experiments using *Ldlr^−/−^* mice, the absence of *TET2* led to significantly larger atherosclerotic lesions compared to control mice, indicating that CHIP mutations enhance the severity of atherosclerosis [4,8]. Notably, these mutations did not cause leukocytosis, meaning that the effect was not due to an increased number of circulating immune cells but rather qualitative changes in immune cell function, especially myeloid cells. Further studies also demonstrated that *TET2* and *DNMT3A* loss exacerbate HF by promoting cardiac fibrosis, and hypertrophy, and reducing cardiac function. This underscores the role of altered immune cell behavior in CHIP-associated heart disease. Additionally, *JAK2* mutations, specifically *JAK2^V617F^*, have been shown to promote thrombosis and atherosclerosis in mouse models [4,10,13]. In these cases, the formation of NETs leading to blood clot formation generally increased inflammation, and hyper-activation of platelets and coagulation pathways were contributing factors [14] (Figure 2). Even in the presence of lower cholesterol levels, mice with *JAK2* mutations developed accelerated atherosclerosis, suggesting that these mutations act as potent drivers of CVD. As was mentioned earlier in the article, direct evidence linking ASXL1 mutations to endothelial dysfunction is limited, but the pro-inflammatory environment induced by these mutations can adversely affect endothelial cells. The inflammatory milieu driven by ASXL1 mutations, marked by elevated cytokines like TNF-α, may indirectly contribute to endothelial dysfunction and increased cardiovascular risk [15].

A human study found that CHIP mutations were found in 19% of participants with coronary endothelial dysfunction (CED) and 13% in those without CED [16]. The presence of CHIP was associated with increased levels of inflammatory markers IL-6 and IL-8 in participants with CED, suggesting a link between CHIP and heightened inflammation [16]. Participants with CHIP mutations had a significantly higher risk of major adverse cardiovascular events (MACEs), particularly those with CED, even when accounting for variables like age, sex, and comorbidities [16]. The study suggests CHIP may contribute to early vascular aging and sustained cardiovascular risk, marked by inflammation in patients with endothelial dysfunction.

### 3.2. Unraveling the Genetic Mutations

As humans age, a steady increase in somatic mutations occurs across various tissues, including hematopoietic stem cells (HSCs). These mutations result from processes like spontaneous deamination of 5-methylcytosine and the error-prone repair of DNA double-strand breaks. On average, each HSC acquires around 170 mutations per decade [4,17]. While most of these mutations do not affect cell fitness, occasionally, a mutation provides a selective advantage, leading to the expansion of a particular blood cell clone. There are several somatic mutations of varying frequency linked to clonal hematopoiesis [18]. Most of the instances of CHIP are caused by *DNMT3A*, *TET2*, and *ASXL1,* which are grouped as DTA genes; however, they are not the only CHIP mutations [18]. In a study of 97,691 genomes, the researchers identified 4938 CHIP mutations across 4229 individuals [18]. The median VAF of these mutations was 16%. Consistent with previous studies, over 75% of these mutations occurred in these three key DTA genes [18]. Around 15% of the mutations were found in the next five most frequently affected genes: *PPM1D*, *JAK2*, *SF3B1*, *SRSF2*, and *TP53* [18]. Significant variability in clonal fraction was observed among these eight genes [18]. For instance, the clonal fraction in peripheral blood was approximately 25% smaller for *DNMT3A* (*p* = 1.3 × 10^−15^) and 14% smaller for *TET2* (*p* = 2.1 × 10^−4^) compared to *ASXL1*, suggesting gene-specific differences in clonal selection [18]. Notably, 90% of individuals with CHIP driver mutations had only a single identified mutation [18]. CHIP prevalence was strongly correlated with age, and smoking increased the odds of developing CHIP by 18%. *JAK2* mutation carriers were the youngest, while carriers of *PPM1D*, *SF3B1*, and *SRSF2* mutations were older by 5 to 7.7 years on average [18].

*DNMT3A* (DNA methyltransferase 3A) encodes a methyltransferase that catalyzes DNA methylation, which is crucial for regulating gene expression [19]. Pathogenic mutations in *DNMT3A* are typically loss-of-function, including missense mutations in its catalytic domains, nonsense mutations, and indels, leading to enhanced self-renewal of HSCs [20]. These mutations promote the expression of multipotency genes and suppress differentiation genes, enabling *DNMT3A* mutations to affect various hematopoietic lineages, contributing to pro-inflammatory T-cell polarization and activation of the inflammasome complex [21]. Experimental studies using CRISPR-modified mouse models show that *DNMT3A* mutations result in aberrant inflammation [22]. *DNMT3A*-mutant HSCs outcompete wild-type cells under chronic infection conditions, leading to clonal expansion due to resistance to apoptosis and differentiation defects [22]. This inflammation-driven clonal expansion is linked to cardiovascular consequences, such as cardiac hypertrophy and fibrosis [22]. In human studies, *DNMT3A* mutations are associated with elevated expression of inflammatory cytokines and T-cell activation, contributing to cardiovascular pathologies like aortic stenosis and heart failure (HF) [23,24].

*TET2* (ten-eleven translocation-2) plays a critical role in DNA demethylation by oxidizing methyl groups, functioning oppositely to *DNMT3A* [19]. Despite these biochemical differences, *TET2* mutations also enhance HSC self-renewal and lead to myeloid lineage bias [25]. *TET2*-deficient macrophages are highly pro-inflammatory, and this state accelerates atherosclerosis, as demonstrated in mouse models with increased atherosclerotic plaque burden and promotion of myocardial fibrosis, HF, and worsened cardiac outcomes in response to stress [26]. In human studies [23], *TET2* CHIP carriers have elevated circulating inflammatory markers, including non-classical monocytes that secrete TNF-α, IL-1β, and IL-8.

*ASXL1* (additional sex combs-like 1) plays a key role in regulating polycomb-mediated transcriptional repression. *ASXL1* mutations drive myeloid transformation [27] but also impair HSC function in mice, leaving the precise mechanisms behind *ASXL1*-induced clonal hematopoiesis unclear. Furthermore, the relationship between *ASXL1* mutations and inflammation or atherosclerosis remains poorly understood, though it is hypothesized that the myeloid bias seen in *ASXL1* CHIP carriers may produce effects similar to those seen with *TET2* loss-of-function mutations [19].

*JAK2* (Janus kinase 2) is a non-receptor tyrosine kinase that plays a crucial role in hematopoietic signaling by transmitting signals downstream of cytokine receptors [19]. It activates *TET2* via tyrosine phosphorylation in response to cytokines, thereby linking extracellular signals to epigenetic regulation of hematopoiesis [28]. *JAK2^V617F^* gain-of-function mutations are closely linked to myeloproliferative neoplasms like essential thrombocytopenia and myelofibrosis, which significantly elevate the risk of thrombotic events, including stroke and deep vein thrombosis (*p* = 0.0003, compared to non-CHIP) [13]. These mutations promote thrombosis through neutrophil extracellular trap formation, with studies showing increased incidence of deep vein thrombosis and pulmonary embolism in *JAK2^V617F^* CHIP carriers [13]. Elevated levels of pro-inflammatory cytokines, particularly IL-18 and IL-6, further exacerbate inflammation [29]. *JAK2^V617F^* mutations also confer the highest coronary artery disease (CAD) risk among CHIP variants, with up to a 10-fold increase [30]. In mouse models, *JAK2^V617F^* mutations accelerate atherosclerosis, macrophage proliferation, and oxidative damage, contributing to fibrosis and HF [31].

*TP53* (tumor protein p53) and *PPM1D* (protein phosphatase 1D) are DNA repair genes. *PPM1D* plays a key role in the DNA damage response pathway and interacts in a feedback loop with the tumor suppressor *TP53* [19]. When *TP53* is activated, it induces *PPM1D* expression, which in turn downregulates apoptosis by dephosphorylating p53 [19]. *PPM1D* loss-of-function mutations are particularly associated with CH after exposure to chemotherapy agents such as cisplatin, etoposide, and doxorubicin [32]. There is an association between *TP53*-mutant CHIP and atherosclerotic diseases like CAD and peripheral artery disease (PAD) [11]. Experimental mouse models demonstrated that p53-deficient CHIP leads to larger atherosclerotic plaques with increased macrophage accumulation, suggesting that *TP53* mutations directly accelerate atherosclerosis. Unlike mutations in genes like *TET2* or *DNMT3A*, *TP53*-CHIP did not increase pro-inflammatory cytokines such as IL-1β and IL-6, indicating different mechanisms of atherogenesis.

Lastly, *SF3B1* and *SRSF2* are both crucial components of the mRNA spliceosome [19]. Defects in these genes disrupt the splicing and export of mRNAs that control translation processes [19]. Though their role in cardiovascular disease is not well-studied, elevated circulating IL-18 levels have been observed in patients with *SF3B1*-mutant CHIP [18].

When examining the association of specific CHIP driver mutations with the risk of stroke, researchers have found that mutations in *DNMT3A*, *TET2*, *ASXL1*, *JAK2*, and *TP53* were present at varying frequencies, with *TET2* being significantly associated with an increased risk of total stroke (HR, 1.93; *p* = 0.006) and IS (HR, 1.93; *p* = 0.006). For HS, both *TET2* (HR, 1.50; *p* = 0.15) and *DNMT3A* (HR, 1.44; *p* = 0.03) showed similar effect sizes, with TET2 having a slightly higher hazard ratio [5].

### 3.3. Epigenetics and CHIP

As mentioned above, a few of the most prevalent mutated genes of CHIP are epigenetic regulators, such as *DNMT3A*, *TET2*, *ASXL1*, and *JAK2* [19]. *DNMT3A* and *TET2* regulate opposing enzymatic functions essential for DNA methylation. *DNMT3A* promotes the production of a DNA methyltransferase enzyme, which adds methyl groups to CpG sites, altering gene expression and acting as a tumor suppressor by silencing specific genes. In contrast, *TET2* produces a DNA demethylase, which removes these methyl groups and recruits histone deacetylases to promoters, effectively reversing the epigenetic changes caused by *DNMT3A*. *ASXL1* is responsible for epigenetic modulation and regulation of chromatin-binding proteins. However, its function is relatively unknown. The NHGRI-EBI GWAS (genome-wide association study) catalog [26] and MRC-IEU EWAS (epigenome-wide association study) catalog [27] were used to search for stroke-causing genes and the corresponding epigenetic markers. The term “ischemic stroke” was employed as the primary search criterion within these databases. The identified gene sets from both catalogs were manually compared to identify common genes relevant to CHIP and IS. Between two catalogs, four overlapping genes were found in association with IS, as shown in the Table 1 below. Among these, ZFHX3 and SH2B3 have already been established as linked to stroke. Yet, the connection between the expression of the remaining genes, stroke, and other CVDs remains unclear, as discussed below. To bridge this gap, the role of each gene in the context of CVDs is reviewed and described, offering insights into their potential as future stroke biomarkers.

*ZFHX3* (zinc finger homeobox 3) has been associated with stroke, particularly atrial fibrillation (AF), which is a significant risk factor for IS [40]. Variants in the *ZFHX3* gene have been linked to increased susceptibility to AF, thus elevating the risk of stroke due to embolic events. Studies have identified single nucleotide polymorphisms (SNPs) within *ZFHX3* that are significantly associated with both AF and IS, underscoring the gene’s role in the development of cerebrovascular diseases [41]. Given its involvement in cardiac conduction and AF, *ZFHX3* is considered a genetic marker for stroke risk, particularly concerning arrhythmia-induced events [40].

The *SH2B3* gene, which encodes the lymphocyte adaptor protein LNK, has been associated with an increased risk of stroke, particularly due to its role in regulating inflammation and immune responses [42]. Variants like rs3184504 in *SH2B3* are linked to pro-inflammatory pathways and the dysregulation of cytokine signaling, which can contribute to endothelial dysfunction, thrombosis, and vascular complications. LNK’s negative regulation of these pathways helps maintain vascular integrity, and its deficiency or mutation may predispose individuals to ischemic stroke through enhanced inflammation and clot formation [43].

The *DNM2* gene (Dynamin 2) plays an important role in various cellular processes, including vesicle trafficking and endocytosis. While there is growing interest in *DNM2′s* role in blood and hematopoietic systems, especially in clonal hematopoiesis and myelodysplastic syndromes, the direct link between *DNM2* mutations and stroke is not yet well-established in the scientific literature. There is, however, indirect evidence that *DNM2*-related mechanisms could be involved in processes relevant to stroke. *DNM2* mutations can affect blood cell formation and immune response regulation, including neutrophil migration, which could play a role in vascular inflammation—a key factor in stroke development [44]. Moreover, *DNM2* has been shown to influence endothelial cell function, which is important for maintaining vascular integrity and could, in theory, affect stroke risk.

*SETBP1* has been associated with CHIP, a condition linked to an increased risk of cardiovascular diseases, including stroke. In a study investigating stroke outcomes, *SETBP1* mutations were identified in stroke patients, with CHIP carriers exhibiting distinct profiles of cardiovascular risk [45]. While the direct role of *SETBP1* in stroke pathogenesis is still being studied, its involvement in clonal hematopoiesis suggests that it may contribute to an increased risk of ischemic stroke through mechanisms involving inflammation and vascular dysfunction.

There are several studies showing this effect of CHIP on epigenetic age, such as a study by Soerensen et al. on elderly Danish twins [46]. Unlike previous research that treated CHIP as a uniform entity, this study distinguishes between mutations in specific genes like *TET2* and *DNMT3A* and their divergent effects on different types of epigenetic aging. For instance, *DNMT3A* mutations show a stronger association with intrinsic epigenetic age acceleration (IEAA), which relates to the biological aging of cells, while *TET2* mutations correlate more with extrinsic age acceleration, which reflects immune system aging. This gene-specific differentiation provides more nuanced insights into the behavior of CHIP and its influence on aging pathways. Additionally, *TET2* mutations tend to arise later in life than *DNMT3A* mutations, possibly due to the different environments needed for clonal expansion, with *TET2*-associated clones thriving in immunologically aged or senescent settings. The study [46] also highlights the potential for CHIP to be used as a tool for identifying individuals at elevated risk for adverse outcomes, such as heart disease or mortality. However, its findings diverge from those of other studies, such as Nachun et al. [47], which suggested that combining CHIP and accelerated epigenetic aging (e.g., AgeAccelHG) could help stratify high-risk individuals. This discrepancy may arise from differences in cohort sizes, age distributions, and methylation data normalization methods. While this study did not replicate the exact results from Nachun et al., it still underscores the importance of considering age and CHIP mutations in clinical stratification models, particularly for identifying at-risk elderly individuals. As expected, the latter study also demonstrated that CHIP accelerates epigenetic aging and shows strong associations with intrinsic epigenetic aging clocks such as Horvath and IEAA, likely due to shared genetic factors, such as polymorphisms near *TERT* and *TRIM59* [47]. One of the most important findings is that combining CHIP with high scores in epigenetic aging clocks, such as Hannum and GrimAge, creates a potent risk factor for adverse health outcomes, including CHD and overall mortality. Individuals with both CHIP and high AgeAccelHG scores face a synergistic increase in risk, while CHIP without epigenetic aging shows no substantial association with poor outcomes. This context dependence highlights the influence of lifestyle factors (e.g., smoking, diet, BMI) on CHIP’s impact.

### 3.4. Inflammatory Pathways

Most common mutated genes mentioned above are known to cause inflammation, making CHIP a pro-inflammatory condition that leads to diseases such as stroke. CHIP mutations are more commonly found in myeloid cells, natural killer cells, and monocytes, where they drive inflammatory responses [12,48]. *TET2* and *DNMT3A* loss-of-function mutations are known to cause dysregulated T-cell function, which leads to altered polarization of immune responses, promoting inflammation [49,50] (Figure 1B). *TET2* mutations are especially linked to upregulation of pro-inflammatory gene expression in macrophage cells, leading to increased production of IL-1β, IL-6, and chemokines like CXCL1 and CXCL2 [26]. This upregulation is often mediated by increased activation of the NLRP3 inflammasome, which further promotes the production of IL-1β [8]. Similarly, mutations in *DNMT3A* in macrophages exhibit comparable but distinct pro-inflammatory gene expression profiles, reinforcing the inflammatory response driven by CHIP. Shortly, CHIP mutations lead to enhanced inflammation by altering immune cell function and increasing the production of pro-inflammatory cytokines, contributing to conditions like atherosclerosis (Figure 2) and other inflammatory diseases.

Unlike the mutations described above, some CHIP mutations have no established link to stroke; however, it could be speculated based on their inflammatory actions. *ASXL1* leads to the skewed differentiation of monocytes and macrophages, resulting in increased production of pro-inflammatory cytokines such as TNF-α [15]. This upregulation is mediated through the EGR1-TNF-α axis, which enhances inflammation and contributes to disease progression [15]. *JAK2^V617F^* mutant cells exhibit heightened activation of the JAK-STAT1 signaling pathway. Similarly to DNMT3A and TET2 mutations, this enhances transcription of pro-inflammatory cytokines, including IL-6, IL-1β, and TNF-α. However, a thing unique to this CHIP mutation is *JAK2*-mutant neutrophils being prone to form neutrophil extracellular traps (NETs), contributing to tissue inflammation and thrombotic complications. [31]. Also, macrophages from *JAK^2V617F^* mice displayed heightened inflammasome activation, marked by increased secretion of IL-1β and IL-18. [10]. The roles of *SF3B1* and *SRSF2* mutation in inflammatory pathways are now well-known; however, there were reported cases of elevated circulating IL-18 levels in patients with *SF3B1* mutation [18].

Chronic inflammatory conditions, including various pro-inflammatory and rheumatologic diseases, are associated with CHIP. For instance, systemic sclerosis (SSc) patients show a higher prevalence of CHIP (25%) compared to healthy individuals (4%), with *DNMT3A* mutations being most common, although no significant clinical differences were noted between CHIP and non-CHIP carriers [51]. Rheumatoid arthritis (RA) patients also demonstrate an elevated CHIP prevalence, especially in older patients, with *DNMT3A* and *TET2* mutations prevalent [52]. The association between CHIP and other rheumatologic conditions like systemic lupus erythematosus (SLE) is still uncertain, though these conditions carry an increased risk of myeloid neoplasms. Ulcerative colitis (UC), an inflammatory bowel disease, is also linked to *CHIP*, particularly with *DNMT3A* and *PPM1D* mutations, and is associated with an increased risk of ischemic heart disease. Elevated Th17 cells and related cytokines, important in IBD pathogenesis, are similarly observed in *DNMT3A*-CHIP carriers with severe aortic stenosis. One such mechanism is chronic, low-grade inflammation, known as “inflammaging”, which is characterized by elevated levels of inflammatory markers like IL-6 and C-reactive protein (CRP) in older adults [4]. This inflammatory state is linked to an increased risk of chronic diseases of aging. Recent studies have identified clonal hematopoiesis, where blood cell clones with somatic mutations expand in older individuals, as a potential link between aging, inflammation, and cardiovascular diseases.

## 4. Clinical Implications of Clonal Hematopoiesis in Stroke

### 4.1. Risk Stratification

Stroke ranked as the second leading cause of death and long-term disability worldwide. Approximately 87% of all strokes are ischemic, while around 10% are due to intracranial hemorrhage, and 3% result from subarachnoid hemorrhage [53,54,55]. Incorporating CH into stroke risk stratification models as an independent predictor can enhance predictive accuracy, particularly in older adults, and facilitate more personalized preventive strategies. This approach allows for the identification of high-risk individuals earlier and promotes targeted interventions to prevent adverse outcomes.

Recent studies have incorporated CHIP in risk stratification models, particularly in predicting the progression to hematologic malignancies and understanding cardiovascular risks. For example, the Clonal Hematopoiesis Risk Score (CHRS) was developed to estimate the likelihood of CHIP/CCUS (Clonal Cytopenia of Undetermined Significance) patients progressing to myeloid malignancies [56]. This model leverages data from large cohorts, such as the UK Biobank, to identify and weight key prognostic factors like high-risk mutations (e.g., in *TP53* or *RUNX1*), clone size, and cytopenia. The CHRS then stratifies patients into low, intermediate, or high-risk groups for myeloid malignancies. A similar model could be created with a focus on stroke by adding other biomarkers and risk factors specific to the disease such as hypertension, diabetes, and cholesterol levels.

However, there are current limitations to incorporating CH into routine clinical screening. One of the main challenges is the cost and complexity of genetic testing required for CHIP detection. Technologies like NGS, while highly sensitive, are expensive and not widely accessible in all healthcare settings. Whole-genome and whole-exome sequencing offer comprehensive mutation coverage but are costly (>$1000/sample), while targeted amplicon sequencing and hybrid capture panels are more affordable ($30–$150/sample) but have technical limitations, making them more appropriate for research rather than routine clinical use [57]. This raises questions about the cost-effectiveness of routine CH screening, particularly for individuals without overt cardiovascular disease or other high-risk factors. It is also worth considering that most likely these expenses are not covered by insurance.

The clinical significance of CH, however, is not yet fully understood. While there is evidence linking CH to increased cardiovascular risk, the exact mechanisms remain unclear, and it is uncertain whether interventions targeting CH can meaningfully reduce stroke incidence or improve outcomes. In addition, not all CH mutations carry the same risk, and the presence of a CHIP mutation does not necessarily indicate an imminent cardiovascular event. This variability complicates the decision of whether to incorporate CH screening into standard practice. In light of these uncertainties, routine CHIP screening may not be necessary for the general population. Current evidence supports its potential use in high-risk individuals, such as older adults with existing cardiovascular disease or multiple risk factors, but broader screening may need further validation through large-scale studies before it can be recommended as part of standard preventive care. The decision to screen for CHIP should be weighed against the potential benefits, costs, and current gaps in our understanding of its long-term impact.

### 4.2. Therapeutic Targeting

Mitigating the cardiovascular and stroke risks associated with CH is an emerging area of research, focusing primarily on targeting specific mutations driving CH and anti-inflammatory treatments. FDA-approved therapies, such as IDH1 inhibitors (ivosidenib), IDH2 inhibitors (enasidenib), and *JAK2* inhibitors (ruxolitinib and fedratinib), directly target the underlying mutations [58]. Hypomethylating agents like azacitidine and decitabine have shown increased efficacy in managing *TET2*-mutant myeloid malignancies [59,60], while high-dose vitamin C can limit the expansion of hematopoietic stem and progenitor cells (HSPCs) with *TET2* deletions [61]. Mutations in the *JAK2* gene, particularly *JAK2^V617F^*, are common in CH and are linked to increased thrombotic events. JAK-STAT pathway inhibitors, such as ruxolitinib, could potentially reduce the pro-thrombotic and pro-inflammatory effects of these mutations [14].

Since CH is strongly linked to increased systemic inflammation, which plays a key role in both stroke and CVD pathogenesis, targeting inflammatory pathways offers a promising therapeutic strategy. Studies indicate that blocking IL-6 signaling—another key inflammatory mediator—might reduce CVD risk in patients with large CHIP clones [62]. Mouse models with *TET2* or *DMMT3A* mutations exhibit elevated IL-6 levels, implicating this pro-inflammatory pathway. Canakinumab, an IL-1β inhibitor, has demonstrated efficacy in reducing cardiovascular events by lowering inflammation, as shown in the CANTOS trial, suggesting its potential benefit for CH-positive individuals [62,63]. Targeting IL-1β, upstream of IL-6, reduces CVD events, suggesting IL-6 blockade could be a promising strategy for individuals with CHIP while potentially avoiding adverse effects like elevated LDL cholesterol. Similarly, Colchicine, an anti-inflammatory agent traditionally used for gout, has been effective in reducing CVD risk in trials like COLCOT, by addressing the inflammation that may drive clonal expansion in CH [64].

## 5. Clonal Hematopoiesis as a Biomarker in Stroke

### 5.1. Prognostic Value

Current stroke risk assessment tools, like the Essen Stroke Risk Score (ESRS) and Stroke Prognostic Instrument-II (SPI-II), are only moderately effective, considering factors like age, gender, obesity, and smoking [65]. However, these tools overlook the role of CHIP, which involves mutations in genes like *TET2* and *DNMT3A* that drive chronic inflammation. This inflammation contributes to endothelial dysfunction, atherosclerosis, and increased cardiovascular disease risk, making CHIP a potential biomarker for predicting ischemic stroke risk [65]. Köhnke and Majeti suggest focusing on two high-risk groups when planning prospective trials for CHIP [58]. The first group is individuals with DNMT3A or TET2 mutations, who face elevated cardiovascular disease CVD risks, highlighting the need to determine VAF thresholds for risk assessment. Current evidence indicates that even low VAF levels may correlate with increased CVD mortality, such as VAF ≥ 1.15% for *DNMT3A* and ≥0.73% for *TET2* in IS. Secondly, in cancer patients, CH mutations like *TP53*, *PPM1D*, or *CHEK2* may increase the risk of therapy-related myeloid neoplasms (tMNs), suggesting that prospective trials should evaluate whether chemotherapy or radiotherapy should be avoided in these individuals [58]. Alternative treatment strategies, including avoiding allogeneic stem cell transplants in lymphoma patients with CH mutations, are also being considered. However, they also discuss how designing clinical trials for CH involves several challenges [58]. The low risk of CH progression in many cases means that treatment-related toxicities must be carefully balanced against the risk of disease progression. Additionally, the long latency between CH detection and events like cardiovascular disease or myeloid malignancies makes trials based on primary endpoints challenging. Surrogate markers, such as reductions in mutant VAF or decreases in mutant clones, may need to be used, though their correlation with long-term outcomes is unclear. Trials must also address difficulties like identifying asymptomatic patients and determining appropriate treatment durations. If treatments are approved, CH would become a public health issue, requiring large-scale genetic screening in primary care, demanding new infrastructure and coordination across clinical labs, primary care, and public health systems. Trials should initially focus on high-risk groups with the most clinically relevant endpoints.

Data from the previous studies [2,11] can be applied to create predictive models for stroke outcomes based on initial CHIP levels. For example, Jaiswal et al. [2] found that individuals with somatic mutations, particularly those associated with CHIP, show a higher cumulative incidence of ischemic stroke over time. This is significant, as patients with prevalent CHIP mutations are at a 2.6-fold (95% confidence interval, 1.3 to 4.8; *p* = 0.003) higher risk of IS compared to those without mutations [2]. Additional factors like hypertension, age, and diabetes further compound this risk, making CHIP a potential early marker for CVD. By incorporating variables like CHIP status (e.g., presence or absence, VAF < 10%, VAF ≥ 10%), age, hypertension, and diabetes, multivariable models can predict stroke incidence and progression. The studies’ hazard ratios, especially for larger CHIP clones, offer insights into the vascular risks. Competing risk regression models, accounting for comorbidities, could then predict stroke outcomes, and these models would need to be validated in independent cohorts.

### 5.2. Monitoring Disease Progression

Monitoring CH mutations over time in stroke patients could reveal early warning signs of clonal expansion, which is linked to increased inflammation and thrombotic events. This monitoring helps predict recurrent stroke risk, as patients with CHIP often experience worse outcomes due to systemic inflammation, driven by pathways like IL-6 and IL-1β. Detecting early changes in these pathways provides insight into vascular deterioration. Additionally, CH monitoring can inform treatment strategies for related conditions such as coronary artery disease, where clonal expansion destabilizes plaques, increasing heart attack or secondary stroke risks. Tracking these changes supports precision medicine approaches: rapid clonal expansion may necessitate aggressive interventions, while stable clones might suggest conservative management. This approach parallels cancer prognostic studies, where long-term mutation tracking informs disease progression and guides personalized care.

## 6. Conclusions

Somatic mutations, or clonal hematopoiesis of indeterminate potential (CHIP), significantly impact ischemic stroke and cardiovascular diseases. Recent studies emphasize the need for close monitoring of patients with substantial somatic mutations due to their high risk of mortality and morbidity. Our research underscores the critical importance of risk stratification in these patients. Understanding the mechanistic role of somatic mutations in stroke and cardiovascular conditions using/integrating various multi-omics approaches is essential for improving patient outcomes.

## Figures and Tables

**Table 1:** Overlapping Genes Associated with Ischemic Stroke from GWAS and EWAS Catalogs.

Gene	CpG	Location	*p* Values	Study
ZFHX3	cg07786668	chr16:73092391	1.6 × 10^−18^, 3.1 × 10^−6^	[33,34,35,36]
	cg00614832	chr16:73092394	3.2 × 10^−16^, 6.4 × 10^−7^	
SH2B3	cg12885832	chr12:111843885	3.5 × 10^−8^, 2.4 × 10^−14^	[36,37,38]
DNM2	cg25487903	chr19:10828950	4.7 × 10^−6^, 1.7 × 10^−8^	[36,39]
SETBP1	cg20127035	chr18:42260234	9.8 × 10^−7^	[36,40]
	cg00037763	chr18:42281763	3.1 × 10^−6^	

## Data Availability

Not applicable.
